# Noninvasive predictive value of preoperative perfusion index for intraoperative hemodynamic fluctuations in patients undergoing lower-extremity orthopedic surgery under regional anesthesia: a retrospective cohort study

**DOI:** 10.3389/fsurg.2026.1910347

**Published:** 2026-08-12

**Authors:** Heying Fang, Chen Chen, Cancan Wang

**Affiliations:** Department of Anesthesiology, Wuchang Hospital Affiliated to Wuhan University of Science and Technology, Wuhan, China

**Keywords:** anesthesia, hemodynamic monitoring, intraoperative monitoring, lower extremity, orthopedics, peripheral perfusion

## Abstract

**Objective:**

To investigate the predictive value of the preoperative perfusion index (PI) for intraoperative hemodynamic fluctuations in patients undergoing lower extremity surgery.

**Methods:**

A retrospective analysis was conducted on 247 patients who underwent orthopedic lower extremity surgery at Wuchang Hospital from May 2022 to September 2024. Based on the occurrence of intraoperative hemodynamic fluctuations, patients were divided into a hemodynamically unstable group (*n* = 87) and a hemodynamically stable group (*n* = 160). Clinical data and preoperative PI levels were compared between the two groups. Multivariate logistic regression analysis was used to identify factors significantly associated with hemodynamic fluctuations. The predictive value of these factors was assessed using receiver operating characteristic (ROC) curves with DeLong's test, followed by internal validation. Decision curve analysis (DCA) was performed to evaluate clinical net benefit. Patients were additionally divided into high- and low-PI groups based on the optimal cutoff value derived from the ROC curve.

**Results:**

Among the 247 patients, 87 (35.22%) experienced intraoperative hemodynamic instability. Compared with the hemodynamically stable group, the unstable group had significantly lower preoperative PI levels (2.52 ± 1.02 vs. 3.96 ± 1.41, *P* < 0.05). ROC analysis showed that preoperative PI predicted intraoperative hemodynamic fluctuations with an area under the curve (AUC) of 0.798, an optimal cutoff value of 3.28, a sensitivity of 80.46%, and a specificity of 69.37%. Using this cutoff, the incidence of hemodynamic fluctuations was significantly higher in the low-PI group (≤3.28) than in the high-PI group (58.82% vs. 13.28%, *P* < 0.05), with a relative risk of 4.43 (95% CI: 2.78–7.07). Multivariate logistic regression analysis identified elevated preoperative heart rate (OR = 1.080, 95%CI: 1.031–1.132), diabetes mellitus (OR = 7.652, 95%CI: 2.295–25.515), and hypertension (OR = 4.325, 95%CI: 1.446–12.937) as independent risk factors for hemodynamic fluctuations. In contrast, elevated preoperative mean arterial pressure (OR = 0.839, 95%CI: 0.778–0.905), elevated preoperative diastolic blood pressure (OR = 0.803, 95%CI: 0.738–0.873), elevated preoperative systolic blood pressure (OR = 0.896, 95%CI: 0.854–0.939), and elevated preoperative PI (OR = 0.296, 95%CI: 0.182–0.483) were protective factors. The combined prediction model based on preoperative PI achieved an AUC of 0.968 (95%CI: 0.937–0.986) for predicting intraoperative hemodynamic fluctuations, which was significantly superior to PI alone (all *P* < 0.001). After 1,000 bootstrap resampling for internal validation, the bias-corrected AUC was 0.954 (95%CI: 0.917–0.986). DCA confirmed that the combined model based on preoperative PI provided a favorable clinical net benefit within the threshold range of 0.1–0.9.

**Conclusion:**

Preoperative PI can effectively predict intraoperative hemodynamic fluctuations in patients undergoing lower extremity surgery. When combined with hemodynamic parameters and underlying comorbidities, preoperative PI offers significantly enhanced predictive performance and clinical net benefit for hemodynamic fluctuations.

## 1 Introduction

Knee and hip arthroplasty are primarily performed to treat end-stage joint diseases or select fractures, including severe osteoarthritis, joint dysplasia, degenerative osteoarthropathy, avascular necrosis of the femoral head, and femoral neck fractures in elderly patients (1). These procedures represent the most mature and commonly performed types of lower extremity joint replacement, and their annual volume continues to rise (2). Nevertheless, as major surgeries, joint replacements carry a non-negligible risk of significant complications. Elderly patients often present with comorbidities such as hypertension and diabetes, making them particularly vulnerable to intraoperative hemodynamic fluctuations, which increase surgical risk and adversely affect postoperative recovery (3). Therefore, identifying reliable predictors of intraoperative hemodynamic fluctuations is of critical importance for both patients and clinicians.

Various methods have been used to predict hemodynamic fluctuations (e.g., intraoperative hypotension), including heart rate variability (4), passive leg raising tests (5), ultrasound measurement of the inferior vena cava and the inferior vena cava-to-aorta diameter ratio (6), echocardiography (7), and the perfusion index (PI) (8). The noninvasive perfusion index (PI) quantifies the ratio of pulsatile to non-pulsatile components in the arterial circulation (9). PI provides clinicians with detailed information on peripheral tissue perfusion dynamics, facilitating the assessment of hemodynamic status, vasomotor response, and early detection of circulatory impairment (10, 11). However, existing studies evaluating the ability of PI to predict hemodynamic fluctuations have predominantly focused on patients undergoing cesarean section or those with intraoperative hypotension (11, 12), while research specifically addressing hemodynamic fluctuations during lower extremity surgery remains limited. Accordingly, the primary objective of this study was to evaluate the predictive value of preoperative PI for intraoperative hemodynamic fluctuations in patients undergoing lower extremity surgery and to determine its optimal cutoff value. This study aims to support early identification of high-risk patients and the implementation of targeted perioperative management strategies.

## 2 Materials and methods

### 2.1 Study subjects

A retrospective analysis was conducted on 247 patients who underwent orthopedic lower extremity surgery at Wuchang Hospital from May 2022 to September 2024. Inclusion criteria were as follows: (1) age ≥18 years; (2) undergoing orthopedic lower extremity surgery (including total knee arthroplasty and total hip arthroplasty); (3) American Society of Anesthesiologists (ASA) I, II, or III; (4) availability of complete clinical data. Exclusion criteria were: (1) emergency surgery; (2) absence of a standardized intraoperative hemodynamic monitoring protocol; (3) preexisting severe uncontrolled hypertension (systolic blood pressure ≥180 mmHg or diastolic blood pressure ≥110 mmHg); (4) preexisting severe cardiac dysfunction (left ventricular ejection fraction <40%) or cardiogenic shock; (5) preexisting severe liver or renal dysfunction (Child-Pugh class C or end-stage renal disease requiring dialysis). The study protocol was reviewed and approved by the Medical Ethics Committee of Wuchang Hospital (2025-006-01), and the requirement for informed consent was waived.

### 2.2 Sample size calculation

This retrospective study enrolled consecutive patients undergoing orthopedic lower extremity surgery between May 2022 and September 2024. During the design phase, sample size estimation was performed based on the events per variable (EPV) principle to construct a stable multivariate prediction model. Assuming approximately k predictor variables would ultimately be included, each variable required at least 10 positive events (hemodynamic fluctuations), thus necessitating at least *n* = 10k positive events. Based on similar literature (13), the estimated incidence (p) of hemodynamic fluctuations in our study population was approximately 51.48%. Accordingly, the minimum total sample size required was calculated as *N* = n/*p* = (10 × k)/0.5148. For k = 5, N ≈ 97 cases. This study retrospectively collected data from 247 eligible patients, which far exceeds this minimum requirement and is sufficient to support model construction.

### 2.3 Clinical data collection

The following data were collected for all patients from the electronic medical record system: (1) General demographic characteristics: age, sex, body mass index (BMI). (2) Lifestyle factors: smoking history, alcohol consumption history. (3) Medical history: history of diabetes, hypertension, coronary artery disease, chronic pulmonary disease. (4) Preoperative vital signs: systolic blood pressure (SBP), diastolic blood pressure (DBP), mean arterial pressure (MAP), and heart rate (HR), measured as a single resting value obtained while the patient was in the supine resting state after entering the operating room and before the induction of anesthesia. (5) Preoperative PI, measured using a pulse oximeter (YK-84A1, Shanghai Hongkang Medical Devices Co., Ltd., Shanghai, China) placed on the patient's right index finger (with the probe positioned on the upper limb of the non-operative side to eliminate interference from surgical manipulation and tourniquet application on the measurement signal). After entering the operating room and before anesthesia induction, each patient remained in a resting state for at least 5 min, after which the baseline PI value was recorded. PI measurements were performed in the operating room preparation area (with central air conditioning maintaining a constant temperature set at 24–26 °C) to ensure consistent environmental conditions across all patients. It should be noted that local skin temperature at the measurement site was not monitored or controlled in this study. (6) Surgical and anesthetic characteristics: type of surgery (total knee arthroplasty/total hip arthroplasty), operative time, ASA classification, anesthesia grade, estimated blood loss, and intraoperative fluid infusion volume. (7) Perioperative management/interventions: norepinephrine use and tourniquet time. (8) Outcome measure: intraoperative hemodynamic status (stable/unstable), determined based on the intraoperative use of vasoactive agents, substantial fluid resuscitation, or blood transfusion.

### 2.4 Assessment of intraoperative hemodynamic Status

This study employed internationally recognized definitions of intraoperative hypotension and clinical intervention indicators to comprehensively assess hemodynamic status (14, 15). Hemodynamic instability was defined as meeting any of the following criteria: Primary criteria: intraoperative mean arterial pressure (MAP) below 20% of the patient's baseline value with an absolute value < 65 mmHg, lasting more than 5 min; or an absolute MAP < 65 mmHg lasting more than 5 min. Secondary criteria: despite MAP maintained at ≥65 mmHg, the presence of obvious signs of inadequate tissue perfusion (e.g., significantly decreased urine output, elevated lactate) or a pronounced downward trend in blood pressure necessitated active administration of vasoactive agents (dopamine, epinephrine, norepinephrine, etc.) by the clinician to maintain circulatory stability. Exclusion bias note: prophylactic crystalloid infusion alone (without concomitant vasoactive agent use or severe MAP decline) was not considered a criterion for hemodynamic instability, in order to reduce selection bias arising from variations in anesthesiologists’ conservative treatment strategies. Hemodynamic stability was defined as: intraoperative MAP consistently maintained at ≥65 mmHg (or a decline of <20% from baseline) throughout the procedure, without the need for vasoactive agents or additional clinical interventions due to circulatory instability. Based on the above criteria, patients were divided into a hemodynamically unstable group (*n* = 87) and a hemodynamically stable group (*n* = 160).

In this study, intraoperative hemodynamic instability was defined as the occurrence of at least 10 hemodynamic fluctuations meeting the predefined criteria during surgery. This threshold was established to differentiate transient physiological oscillations from persistent pathological states, thereby reducing false-positive classification caused by incidental factors related to variations in anesthetic depth or operative stimuli. Additionally, following the principle of “clinical event priority,” if a patient exhibited fewer than 10 fluctuations but experienced a severe event requiring urgent pharmacological intervention (e.g., use of vasoactive agents) or presenting evidence of organ hypoperfusion (e.g., oliguria, elevated lactate), such cases were separately flagged as “clinically significant hemodynamic events for analysis inclusion.”

### 2.5 Statistical methods

Statistical analyses were performed using SPSS version 22.0. Normality of continuous data was assessed using the Shapiro–Wilk test. Normally distributed data were expressed as mean ± standard deviation, and between-group comparisons were performed using the independent-samples t-test (Welch's *t*-test was applied when variances were unequal). Non-normally distributed data were expressed as median (interquartile range) [M (IQR)], with between-group comparisons conducted using the Mann–Whitney U test. Categorical data were expressed as number (percentage) [n (%)] and compared using the *χ*^2^ test. Ordinal data were compared using the rank-sum test.

Hemodynamic fluctuation status during lower extremity surgery was used as the dependent variable (coded as stable = 0, unstable = 1). Variables with *P* < 0.05 in univariate analysis were considered candidate independent variables. Collinearity diagnostics were first performed using the enter method, and only variables with a variance inflation factor (VIF) < 5 were entered into the multivariate logistic regression analysis. Results are presented as odds ratios (ORs) with 95% confidence intervals (CIs) and *P* values.

Based on the independent predictors identified by multivariate logistic regression, a receiver operating characteristic (ROC) curve for predicting hemodynamic fluctuations during lower extremity surgery was plotted using R software (version 4.5.3), followed by internal validation with 1,000 bootstrap resamples. The area under the curve (AUC) of different ROC curves was compared using DeLong's test. Decision curve analysis (DCA) was used to evaluate the clinical net benefit of the combined model. A *P*-value < 0.05 was considered statistically significant.

## 3 Results

### 3.1 Comparison of baseline characteristics between the two groups

Among the 247 patients who underwent orthopedic lower extremity surgery, 87 (35.22%) experienced intraoperative hemodynamic instability. Compared with the hemodynamically stable group, the unstable group had significantly higher preoperative heart rate (HR) and a significantly higher proportion of patients with diabetes and hypertension (all *P* < 0.05; Table 1). Additionally, the unstable group showed significantly lower preoperative mean arterial pressure (MAP), diastolic blood pressure (DBP), and systolic blood pressure (SBP) (all *P* < 0.05; Table 1).

**Table 1:** Comparison of baseline characteristics between the two groups.

Variable	Hemodynamically unstable group (*n* = 87)	Hemodynamically stable group (*n* = 160)	*χ^2^*/*t/Z*	*P*
Age (years)	66 (62,69)	65 (60,70)	0.663	0.507
Sex(Male/Female)	41/46	89/71	1.633	0.201
BMI (kg/m^2^)	23.58 ± 1.47	23.44 ± 1.96	0.589	0.556
Preoperative MAP (mmHg)	76 (71,80)	84 (76,88)	5.788	＜0.001
Preoperative HR (beats/min)	86 (77,94)	76 (71,87)	4.227	＜0.001
Preoperative DBP (mmHg)	58.63 ± 7.42	66.96 ± 6.56	9.113	＜0.001
Preoperative SBP (mmHg)	106 (100,112)	120 (111,127)	7.996	＜0.001
Smoking history(No/Yes)	39/48	80/80	0.604	0.437
Alcohol consumption history(No/Yes)	44/43	78/82	0.075	0.784
Diabetes(No/Yes)	36/51	99/61	9.552	0.002
Hypertension(No/Yes)	38/49	100/60	8.098	0.004
Coronary artery disease(No/Yes)	45/42	99/61	2.389	0.122
Chronic pulmonary disease(No/Yes)	47/40	89/71	0.058	0.809
Surgery type(Total knee arthroplasty/Total hip arthroplasty)	49/38	92/68	0.032	0.858
ASA classification(I/II/III)	29/31/27	54/61/45	0.307	0.759
Operative time (min)	97.41 ± 21.28	100.63 ± 19.17	1.212	0.227
Estimated Blood Loss (mL)	121.58 ± 15.79	119.85 ± 24.17	0.601	0.548
Intraoperative Fluid Volume (mL)	1,850.58 ± 450.17	1,758.41 ± 380.99	1.702	0.090
Use of Norepinephrine(No/Yes)	44/43	96/64	2.039	0.153
Tourniquet Time (min)	65.28 ± 12.57	63.82 ± 11.93	0.901	0.368

### 3.2 Relationship between preoperative PI level and hemodynamic fluctuations

**Figure 1:**
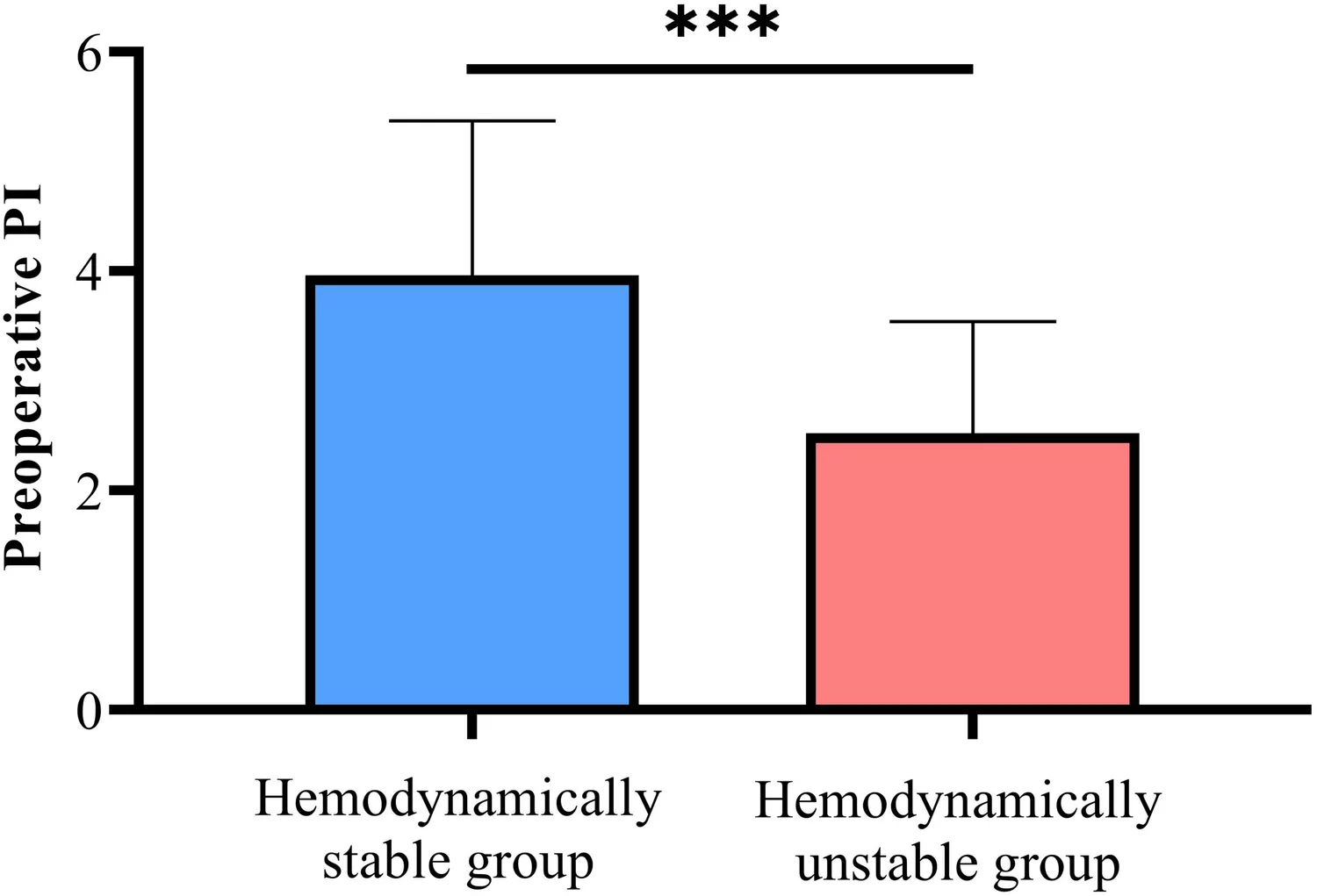
Comparison of preoperative PI levels between patients with different hemodynamic fluctuation statuses.

**Figure 2:**
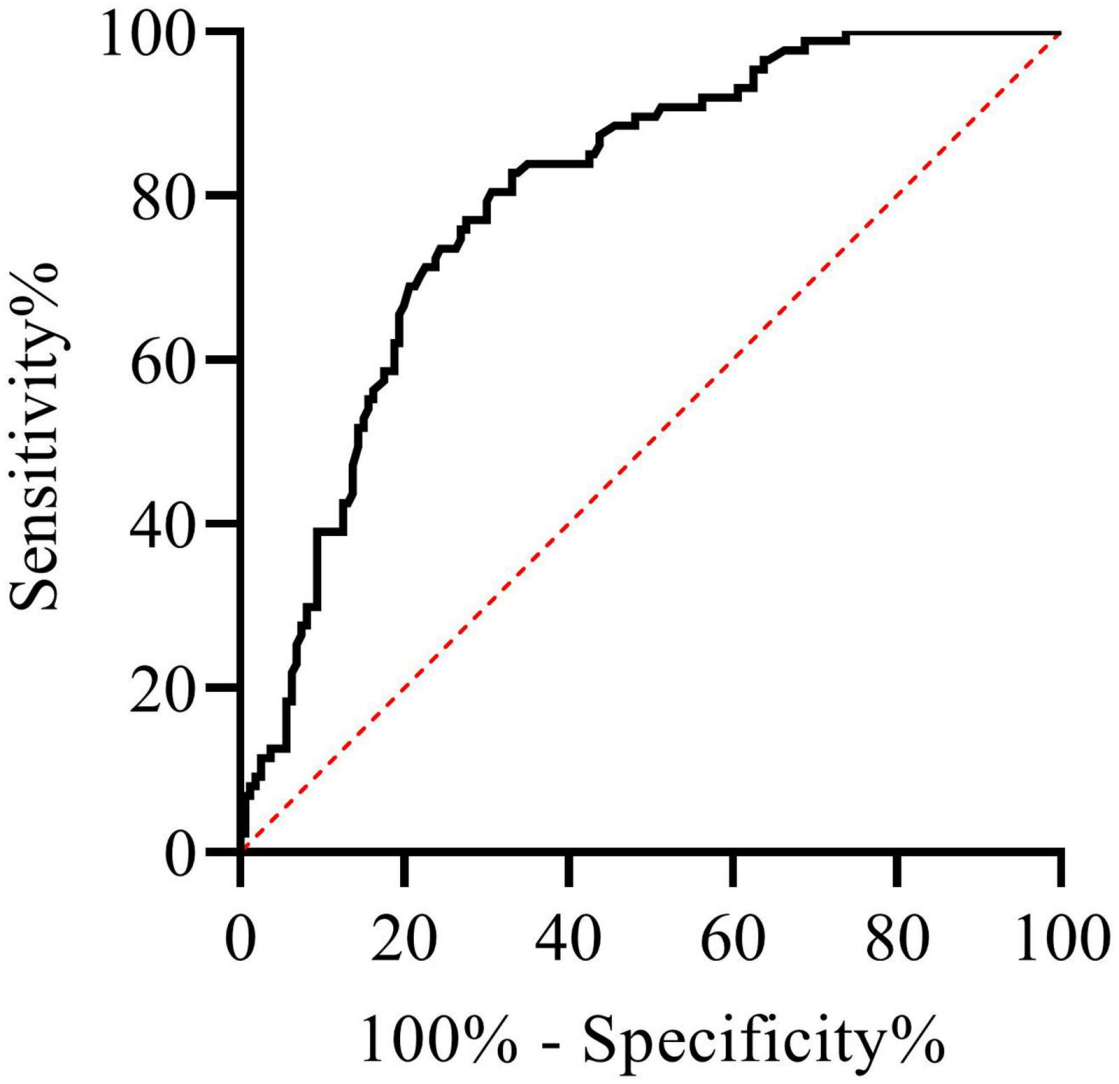
ROC curve of preoperative PI level for predicting hemodynamicfluctuations in patients undergoing lower extremity surgery.

Compared with the hemodynamically stable group, the unstable group had significantly lower preoperative PI levels (2.52 ± 1.02 vs. 3.96 ± 1.41, *P* < 0.05; Figure 1). ROC analysis revealed that the AUC of preoperative PI for predicting hemodynamic fluctuations was 0.798 (Figure 2). Based on the optimal cutoff value derived from the ROC curve (3.28), patients were divided into a high-PI group (>3.28, *n* = 128) and a low-PI group (≤3.28, *n* = 119). The incidence of intraoperative hemodynamic fluctuations was significantly higher in the low-PI group than in the high-PI group (58.82% vs. 13.28%, *χ*^2^ = 56.059, *P* < 0.05). Relative risk analysis indicated that the low-PI group had a 4.43-fold higher risk of hemodynamic fluctuations compared with the high-PI group (RR = 4.43, 95% CI: 2.780–7.073).

### 3.3 Multivariate logistic regression analysis of factors affecting intraoperative hemodynamic fluctuations

Seven variables were included as independent variables: preoperative MAP, preoperative HR, preoperative DBP), preoperative SBP, preoperative PI, diabetes, and hypertension. The dependent variable was intraoperative hemodynamic status. Collinearity diagnostics were first performed using the enter method, and the VIFs for all seven variables were <2 (range: 1.030–1.138). These seven variables were then entered into a multivariate logistic regression analysis using the full model enter method. The results showed that an elevated preoperative HR, diabetes mellitus, and hypertension were risk factors for hemodynamic fluctuations (OR > 1), whereas elevated preoperative MAP, elevated preoperative DBP, elevated preoperative SBP, and elevated preoperative PI were protective factors (OR < 1, Table 2).

**Table 2:** Multivariate logistic regression analysis of factors affecting intraoperative hemodynamic fluctuations in patients undergoing lower extremity surgery.

Variables	*β*	*SE*	Wald *χ^2^*	*P*	*OR*	95%*CI*
Preoperative MAP	−0.175	0.039	20.761	＜0.001	0.839	0.778–0.905
Preoperative HR	0.077	0.024	10.345	0.001	1.080	1.031–1.132
Preoperative DBP	−0.220	0.043	26.556	＜0.001	0.803	0.738–0.873
Preoperative SBP	−0.110	0.024	20.437	＜0.001	0.896	0.854–0.939
Preoperative PI	−1.217	0.249	23.818	＜0.001	0.296	0.182–0.483
Diabetes	2.035	0.614	10.970	0.001	7.652	2.295–25.515
Hypertension	1.464	0.559	6.860	0.009	4.325	1.446–12.937

### 3.4 Predictive value of the preoperative PI-based model for hemodynamic fluctuations

**Figure 3:**
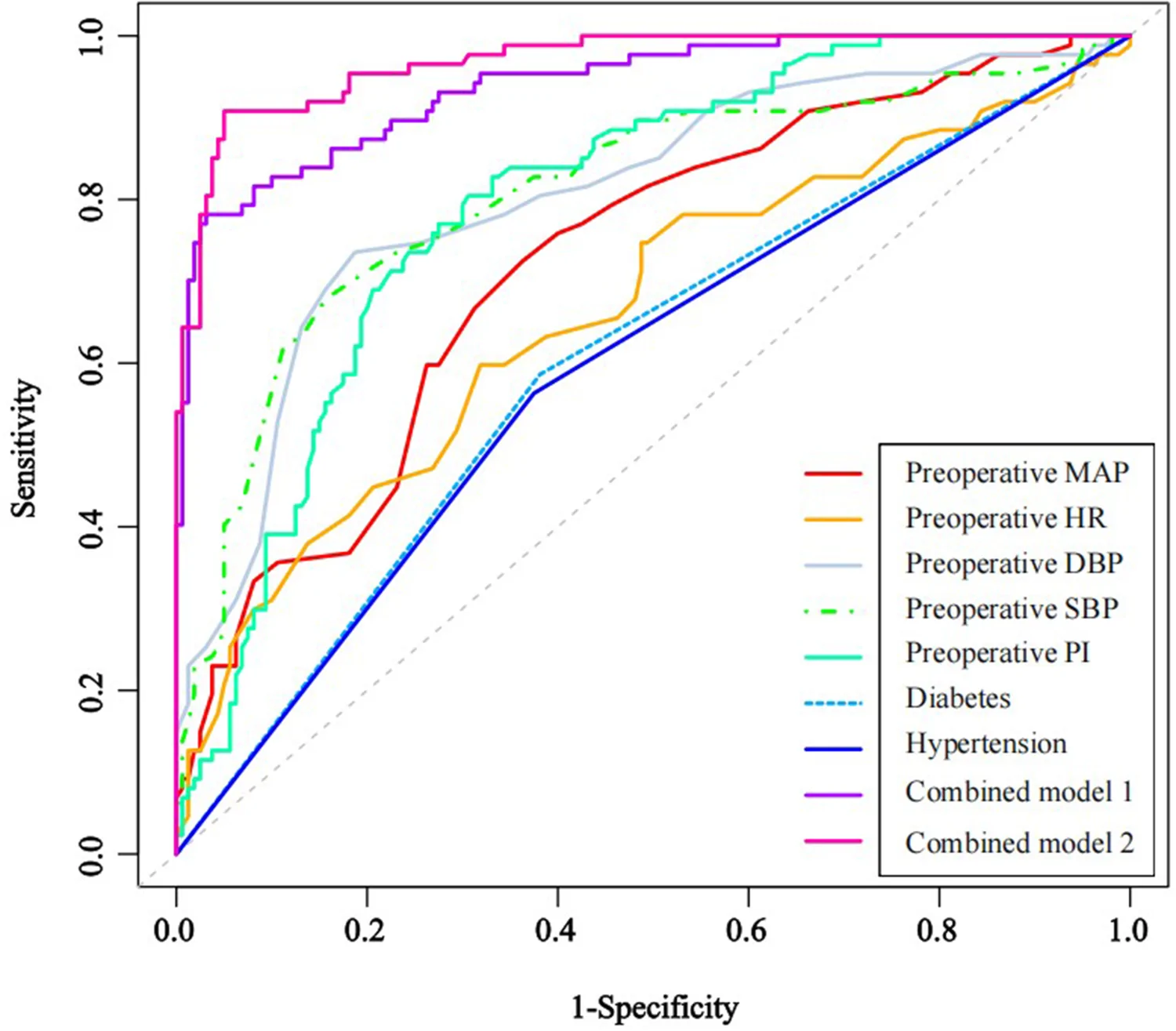
ROC curves of various risk factors for predicting hemodynamic fluctuations in patients undergoing lower extremity surgery.

A combined prediction model excluding preoperative PI (Combined model 1) was constructed using Logistic regression analysis, with the equation: Combined model 1 = 29.247 + (−0.152 × preoperative MAP) + (0.058 × preoperative HR) + (−0.191 × preoperative DBP) + (−0.104 × preoperative SBP) + (1.506 × diabetes) + (1.197 × hypertension). The AUC of Combined model 1 for predicting hemodynamic fluctuations in patients was 0.939 (95% CI: 0.901–0.965, Figure 3).

**Figure 4:**
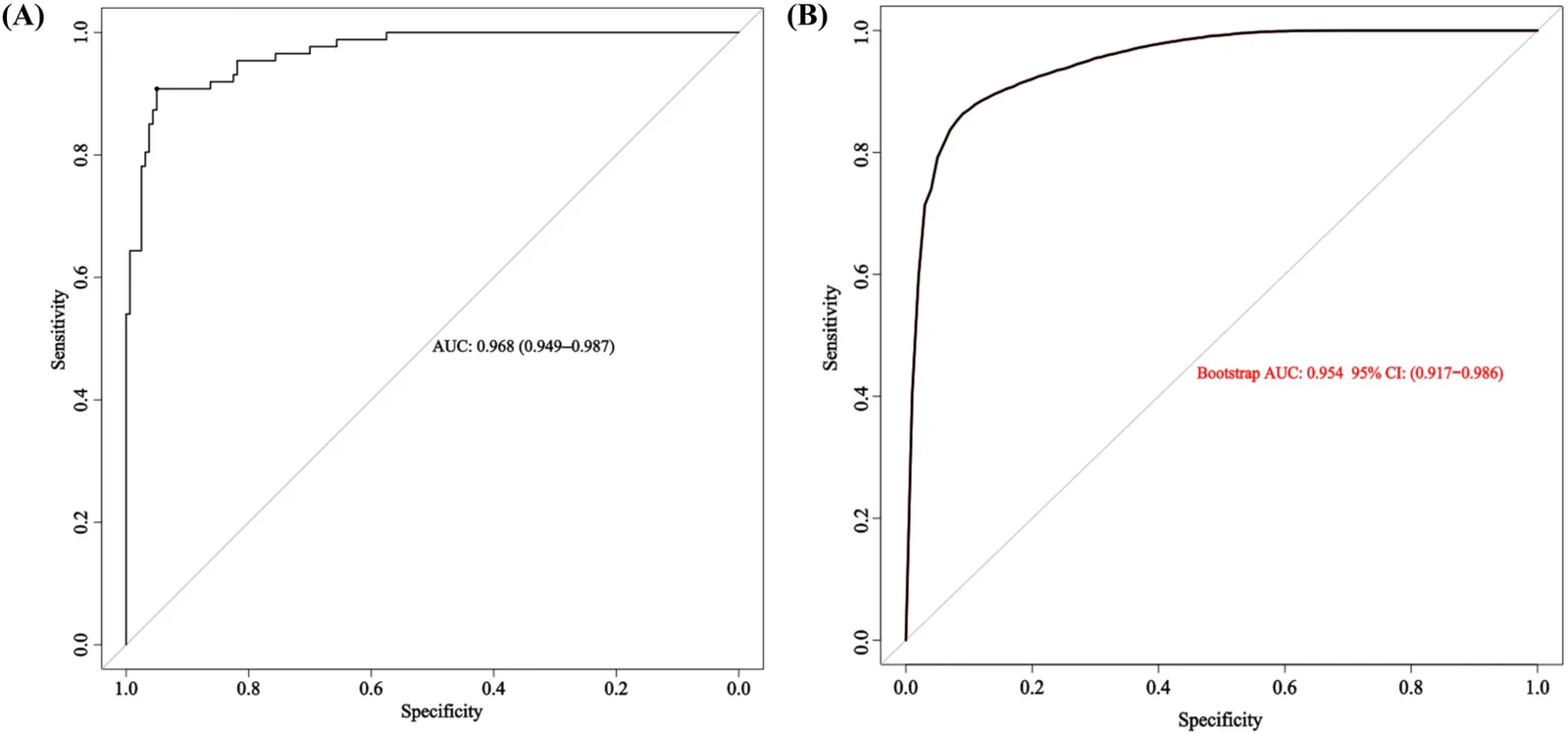
Predictive value of the combined model 2 for hemodynamic fluctuations. **(A)** Original ROC curve. **(B)** Corrected ROC curve.

Furthermore, a combined prediction model incorporating preoperative PI (Combined model 2) was constructed using Logistic regression analysis, with the equation: Combined model 2 = 35.315 + (−0.175 × preoperative MAP) + (0.077 × preoperative HR) + (−0.220 × preoperative DBP) + (−0.110 × preoperative SBP) + (−1.217 × preoperative PI) + (2.035 × diabetes) + (1.464 × hypertension). ROC analysis demonstrated that the original AUC of the combined model 2 for predicting intraoperative hemodynamic fluctuations was 0.968 (Table 3; Figure 4A). The AUC of the combined model 2 was significantly higher than those of preoperative MAP (Z = 7.489, *P* < 0.001), preoperative HR (Z = 8.061, *P* < 0.001), preoperative DBP (Z = 5.718, *P* < 0.001), preoperative SBP (Z = 5.846, *P* < 0.001), preoperative PI (Z = 6.133, *P* < 0.001), diabetes (Z = 10.816, *P* < 0.001), and hypertension (Z = 11.078, *P* < 0.001) when used alone. After 1,000 bootstrap internal validations, the bias-corrected AUC of the combined model 2 was 0.954 (95% CI: 0.917–0.986; Figure 4B).

**Table 3:** Value of preoperative PI for noninvasively predicting hemodynamic fluctuations.

Variables	AUC	Cut off	95%*CI*	Sensitivity (95%*CI*)(%)	Specificity (95%*CI*)(%)	Youden index
Preoperative MAP (mmHg)	0.723	≤79	0.663∼0.778	72.41 (61.62∼81.20)	63.75 (55.74∼71.09)	0.3616
Preoperative HR (beats/min)	0.663	＞84	0.600∼0.721	59.77 (48.69∼69.98)	68.12 (60.23∼75.13)	0.2790
Preoperative DBP (mmHg)	0.806	≤61	0.751∼0.854	73.56 (62.83∼82.18)	81.25 (74.15∼86.81)	0.5481
Preoperative SBP (mmHg)	0.808	≤108	0.753∼0.855	67.82 (56.83∼77.21)	84.37 (77.60∼89.45)	0.5219
Preoperative PI	0.798	≤3.28	0.742∼0.846	80.46 (70.28∼87.89)	69.37 (61.52∼76.28)	0.4983
Diabetes	0.602	——	0.538∼0.664	58.62 (47.55∼68.92)	61.88 (53.84∼69.33)	0.2050
Hypertension	0.594	——	0.530∼0.656	56.32 (45.29∼66.79)	62.50 (54.47∼69.92)	0.1882
Combined model 1	0.939	＞0.594	0.901∼0.965	78.16 (67.77∼86.02)	96.87 (92.48∼98.84)	0.7504
Combined model 2	0.968	＞0.371	0.937∼0.986	90.80 (82.19∼95.66)	95.00 (90.05∼97.66)	0.8580

### 3.5 Decision curve analysis of the combined model

**Figure 5:**
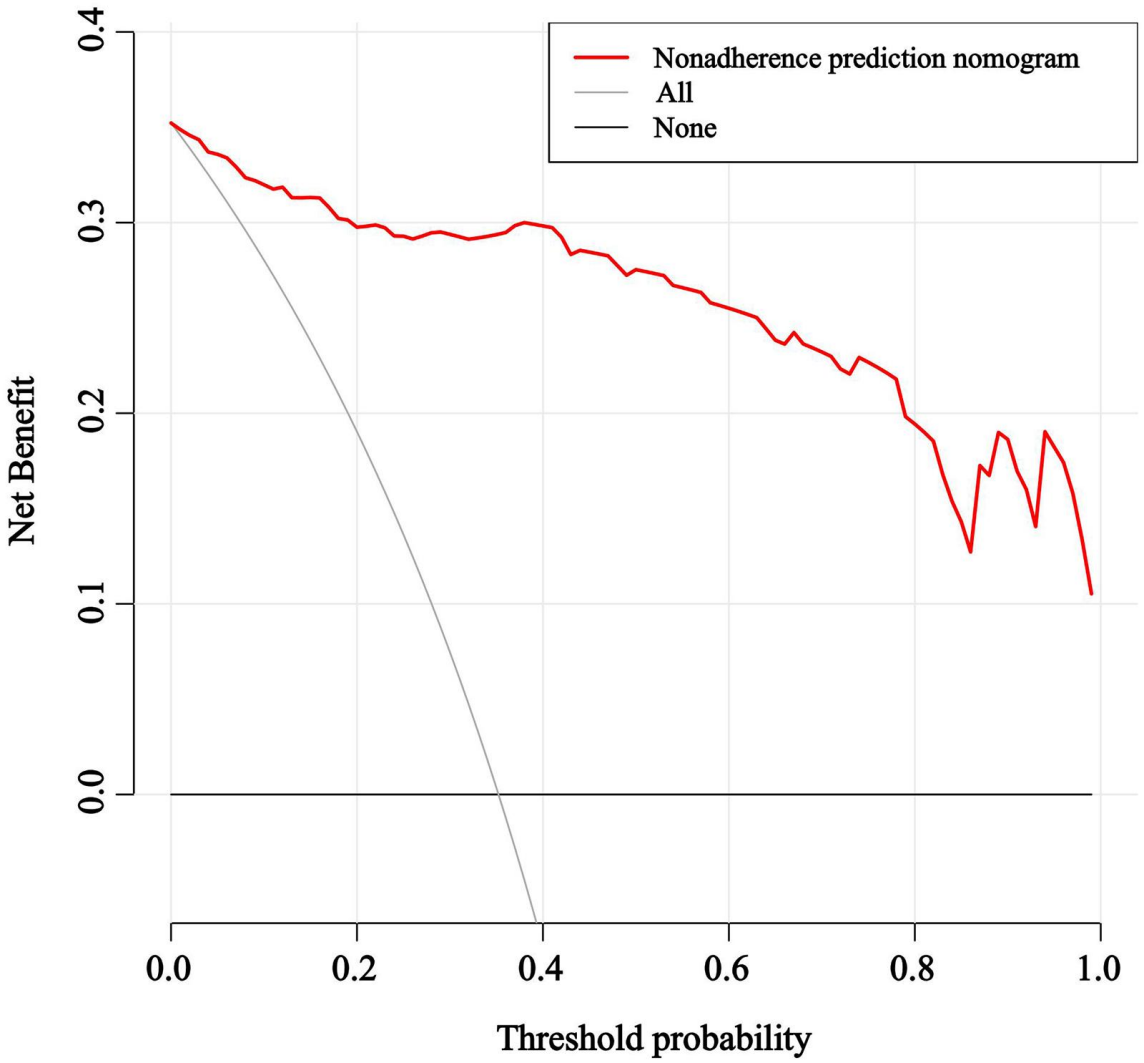
DCA curve of the combined model for predicting hemodynamic fluctuations.

DCA further confirmed that within a threshold probability range of 0.1–0.9, the net benefit of the combined model was higher than that of the “intervention for all” and “intervention for none” strategies (Figure 5).

## 4 Discussion

The present study found that preoperative PI is closely associated with intraoperative hemodynamic stability in patients undergoing lower extremity surgery. Low PI not only reflects preoperative peripheral hypoperfusion and a compensatory high sympathetic tone state but also serves as an important noninvasive indicator for identifying patients at high risk of intraoperative hemodynamic fluctuations. Using a cutoff value of 3.28, preoperative PI demonstrated good predictive performance (AUC = 0.798), and patients with low TPI had a significantly higher risk of hemodynamic fluctuations (RR = 4.43). Thus, PI is not merely a reflection of physiological status but also has clear clinical early warning value.

Low PI indicates that the patient is in a state of high sympathetic tone preoperatively, which often suggests relatively insufficient effective circulating blood volume (16). Following anesthesia induction, sympathetic tone is abruptly suppressed, compensatory mechanisms are removed, and the peripheral vascular bed dilates extensively, leading to a sharp drop in blood pressure that often requires aggressive interventions such as substantial fluid resuscitation or blood transfusion. This explains why patients with low PI in this study were more prone to hemodynamic fluctuations requiring aggressive intervention. These findings are consistent with and differ from several previous studies in certain aspects. For example, Kavak et al. (13) found that preoperative PI (OR: 0.696, 95% CI: 0.514–0.944) was a significant independent protective factor against hypotension during lower extremity surgery. With a preoperative PI cutoff value of 2.25, the sensitivity and specificity for predicting post-spinal anesthesia hypotension were both 65%, and the AUC was 0.675 (95% CI: 0.568–0.781). In contrast, in the study by Duggappa et al. (17), a PI > 3.5 predicted intraoperative hypotension risk with a sensitivity of 82.1%, specificity of 100%, and an AUC of 0.91 (95% CI: 0.81–0.97). Moreover, the incidence of hypotension in the PI > 3.5 group (100%) was significantly higher than that in the PI ≤ 3.5 group (21.9%), suggesting that a baseline PI > 3.5 is a highly specific and sensitive risk predictor for hypotension in elderly patients undergoing spinal anesthesia. The present study also confirms that preoperative PI is an independent predictor of intraoperative hemodynamic fluctuations (e.g., hypotension) and proposes a corresponding predictive cutoff value. However, while Duggappa et al. (17) found that a high PI was associated with an increased risk of hypotension, both Kavak et al. (13) and the present study showed that a low PI is a risk factor for hemodynamic fluctuations. The reasons for this discrepancy may be as follows: (1) Differences in the definition of hemodynamic fluctuations: The present study used “requiring clinical intervention” as the criterion for fluctuations, whereas the aforementioned studies used “absolute blood pressure value/percentage decrease from baseline”. (2) Differences in anesthesia technique: The aforementioned studies focused on patients receiving spinal anesthesia, where the degree of sympathetic blockade is more uniform. Patients with high PI have lower baseline vascular tone and are more sensitive to anesthetic agents, manifesting as a numerical decrease in blood pressure. In contrast, patients with low PI have limited sympathetic compensatory reserve; although their initial blood pressure may be acceptable, they are more prone to decompensation under stress and require pharmacological intervention. (3) Differences in population characteristics: In the study by Kavak et al. (13), preoperative PI showed significant predictive performance in the non-elderly group (<65 years; AUC = 0.727) but failed to predict hypotension in the elderly group (≥65 years; AUC = 0.579, *P* = 0.336), indicating that age significantly influences the predictive value of PI. In comparison, the present study included patients undergoing orthopedic lower extremity surgery with an age range of 47–78 years and did not restrict anesthesia type, resulting in greater population heterogeneity, which may explain the difference in the direction of prediction. Future prospective studies are needed to further validate this hypothesis.

In addition to preoperative PI, this study found that compared with the hemodynamically stable group, the unstable group had significantly higher preoperative HR and significantly lower preoperative MAP, DBP, and SBP. Further ROC curve analysis confirmed that preoperative MAP, HR, DBP, and SBP all had good predictive performance for intraoperative hemodynamic fluctuations in patients undergoing lower extremity surgery. A review by Mukkamala et al. (18) clearly indicated that mean MAP is the most predictive variable for intraoperative hypotension, with a simple MAP-based prediction model (threshold 72–73 mmHg) showing comparable performance to complex hypotension prediction indices. A prospective study by Qi et al. (19) showed that heart rate deceleration capacity measured from a 5-minute preoperative electrocardiogram predicted post-induction hypotension with an AUC of 0.777, and low deceleration capacity (reflecting impaired parasympathetic function) was significantly associated with an increased risk of post-induction hypotension. Although the present study did not assess heart rate variability, elevated preoperative baseline HR similarly reflected a sympathetic-dominated compensatory state. Valadkhani et al. (20) found that an intraoperative absolute DBP < 44 mmHg had the strongest association with perioperative myocardial injury, suggesting that DBP is a sensitive indicator of hemodynamic vulnerability. Building on this, the present study further confirmed that preoperative DBP alone can effectively predict intraoperative hemodynamic fluctuations requiring clinical intervention (AUC = 0.806). Previous studies have mostly focused on the association between intraoperative hypotension and postoperative complications, with fewer reports on the predictive value of preoperative baseline blood pressure levels for intraoperative hemodynamic fluctuations themselves. The present study systematically evaluated the predictive performance of preoperative MAP, HR, DBP, and SBP, confirming that their AUCs were all above 0.65, with DBP and SBP showing particularly outstanding predictive performance. This finding suggests that for patients undergoing lower extremity surgery, routinely measured preoperative baseline hemodynamic parameters can effectively identify those at high risk of intraoperative fluctuations, offering significant clinical application value.

This study also found that the proportions of patients with diabetes and hypertension were significantly higher in the hemodynamically unstable group than in the stable group, and both were significantly associated with an increased risk of hemodynamic fluctuations. However, the predictive performance of diabetes and hypertension alone for hemodynamic fluctuations was relatively weak in this study. This result is highly consistent with several previous studies. For example, Malm et al. (21) found that carotid artery flow parameters were significantly altered in patients with diabetes, characterized by significantly decreased peak systolic velocity, end-diastolic velocity, and mean flow velocity, as well as significantly increased resistive and pulsatility indices. This may be because chronic hyperglycemia in diabetic patients damages the vascular endothelium, leading to increased arterial stiffness and consequent hemodynamic changes. Tunedal et al. (22) used 4D Flow MRI and individualized cardiovascular models to precisely quantify the hemodynamic effects of hypertension and diabetes, demonstrating that the coexistence of hypertension and diabetes imposes an additional load on the left ventricle, leading to diastolic dysfunction that is not apparent in patients with a single disease at rest. This explains why patients in the present study with both comorbidities faced a higher risk of hemodynamic imbalance during lower extremity surgery. It is noteworthy that this study identified hypertension as an independent risk factor for hemodynamic instability, while intraoperative hypotension (manifested as lower SBP, DBP, and MAP) was also closely associated with instability. These findings are not contradictory but rather reflect different pathophysiological dimensions: hypertension, as a chronic condition, is often accompanied by vascular stiffness and blunted baroreceptor reflexes, resulting in diminished compensatory capacity in response to acute hemodynamic perturbations; whereas intraoperative hypotension serves as the direct trigger that disrupts this fragile equilibrium. Consequently, patients with hypertension, due to their impaired baseline regulatory function, may be more susceptible to refractory hypotensive episodes during surgery, thereby increasing the overall risk of hemodynamic instability. In addition, this study observed a relatively high overall rate of vasoactive drug use, which did not fully correspond to the incidence of the hemodynamically unstable group. This phenomenon may be attributed to the fact that regional anesthesia frequently leads to sympathetic blockade and anticipated vasodilation, coupled with the need to maintain a bloodless surgical field in orthopedic lower-limb procedures, prompting anesthesiologists to adopt proactive preventive or early corrective medication strategies. Consequently, although many patients received vasopressors, their blood pressure was rapidly maintained within the normal range and did not meet the stringent criteria for “persistent hypotension” or “inadequate tissue perfusion” defined in this study, and they were thus classified into the “stable group.”

This study further incorporated preoperative PI, preoperative hemodynamic parameters (MAP, HR, DBP, SBP), and underlying comorbidities (diabetes, hypertension) into a multivariate logistic regression model to construct a combined PI-based model. The results showed that the AUC of this combined model for predicting hemodynamic fluctuations was significantly higher than that of preoperative PI alone. Furthermore, the DCA curve indicated that the combined model had a high clinical net benefit within the threshold range of 0.1–0.9. Although diabetes and hypertension are independent risk factors for hemodynamic fluctuations, a single underlying comorbidity has limited predictive value for hemodynamic fluctuations during lower extremity surgery. Therefore, clinical attention should focus more on the synergistic effects of multiple comorbidities or their integration with other physiological parameters for comprehensive assessment.

It should be noted that there are currently multiple approaches to defining hemodynamic instability, including dynamic indicators based on heart rate variability (HRV), blood pressure variability (BPV), as well as absolute blood pressure thresholds or clinical intervention criteria. The present study adopted the latter approach, primarily based on the following considerations: First, this study was positioned as a preoperative static risk stratification, aiming to address the clinical question of “whether a single measurement upon operating room entry can predict intraoperative risk,” rather than exploring the dynamic regulatory mechanisms of autonomic function. Therefore, employing thresholds and intervention criteria that directly correspond to clinical decision-making offers greater practicality. Second, this study was a retrospective analysis, and the medical record system only contained preoperative single resting blood pressure and heart rate values without continuous beat-to-beat data, lacking the data foundation necessary for calculating dynamic indicators such as HRV/BPV. Third, our results demonstrated that univariate indicators based on static baseline values (e.g., preoperative DBP with an AUC of 0.806, preoperative PI with an AUC of 0.798) and the combined model (AUC of 0.968) already exhibited good predictive performance, confirming that this definitional approach is applicable to the present study scenario.

Although this study evaluated the predictive value of preoperative PI and other parameters for intraoperative hemodynamic fluctuations, several limitations should be acknowledged: (1) Preoperative anxiety was not quantified: preoperative psychological stress may reduce PI through sympathetic activation-induced peripheral vasoconstriction, but this study did not perform a quantitative assessment. (2) Lack of core hemodynamic parameters: due to the absence of invasive arterial blood pressure monitoring or central venous catheterization, key data such as systemic vascular resistance and cardiac output were unavailable, limiting the depth of mechanistic exploration. (3) PI was not used to guide intraoperative medication: this study only employed PI as a predictive indicator and did not incorporate it into real-time intraoperative decision-making regarding vasoactive agents. Currently, evidence for PI-guided medication is primarily concentrated in sepsis (threshold ≤ 0.3) (23), and its strategy in patients undergoing neuraxial anesthesia surgery remains to be validated. (4) Insufficient control of environmental temperature: although room temperature was controlled (24–26 °C), local finger skin temperature was not monitored. Future studies should incorporate core-to-peripheral temperature gradient correction for PI to eliminate interference from local temperature variations. (5) Limited external generalizability: the PI cutoff value is influenced by equipment, measurement site, and population characteristics, and should not be directly extrapolated to other clinical settings.

In conclusion, among the patients undergoing lower-extremity orthopedic surgery under regional anesthesia enrolled in this study, preoperative PI was significantly correlated with intraoperative hemodynamic fluctuations, and a lower preoperative PI level was identified as an independent risk factor for intraoperative hemodynamic instability. The predictive model constructed based on preoperative PI combined with baseline vital signs and comorbidities demonstrated high diagnostic performance and significant clinical net benefit.

## Data Availability

The original contributions presented in the study are included in the article/Supplementary Material, further inquiries can be directed to the corresponding author.
